# Microfluidics—A novel technique for high‐quality sperm selection for greater ART outcomes

**DOI:** 10.1096/fba.2024-00041

**Published:** 2024-08-23

**Authors:** Ghulam Rasool Bhat, Farooz Ahmad Lone, Jasmer Dalal

**Affiliations:** ^1^ Division of Animal Reproduction, Gynaecology and Obstetrics Sher‐e‐Kashmir Institute of Agricultural Sciences and Technology of Kashmir Srinagar India; ^2^ Division of Veterinary Gynaecology and Obstetrics Lala Lajpat Rai Veterinary and Animal Sciences University Hisar India

**Keywords:** chips, microfluidics, sperm selection

## Abstract

Microfluidics represent a quality sperm selection technique. Human couples fail to conceive and this is so in a significant population of animals worldwide. Defects in male counterpart lead to failure of conception so are outcomes of assisted reproduction affected by quality of sperm. Microfluidics, deals with minute volumes (μL) of liquids run in small‐scale microchannel networks in the form of laminar flow streamlines. Microfluidic sperm selection designs have been developed in chip formats, mimicking in vivo situations. Here sperms are selected and analyzed based on motility and sperm behavioral properties. Compared to conventional sperm selection methods, this selection method enables to produce high‐quality motile sperm cells possessing non‐damaged or least damaged DNA, achieve greater success of insemination in bovines, and achieve enhanced pregnancy rates and live births in assisted reproduction—in vitro fertilization (IVF) and intracytoplasmic sperm injection (ICSI). Besides, the concentration of sperm available to oocyte can be controlled by regulating the flow rate in microfluidic chips. The challenges in this technology are commercialization of chips, development of fully functional species‐specific microfluidic tools, limited number of studies available in literature, and need of thorough understanding in reproductive physiology of domestic animals. In conclusion, incorporation of microfluidic system in assisted reproduction for sperm selection may promise a great success in IVF and ICSI outcomes. Future prospectives are to make this technology more superior and need to modify chip designs which is cost effective and species specific and ready for commercialization. Comprehensive studies in animal species are needed to be carried out for wider application of microfluidic sperm selection in in vitro procedures.

## Graphical abstract

Sperm selection techniques: special refrence to microfluidics.

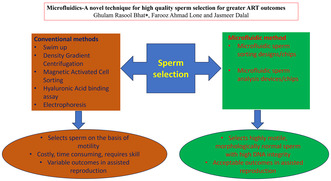

## 1 INTRODUCTION

About 15% of human couples show conception failure naturally and nearly 30% livestock is infertile with male factor contributing 50% to the total.^1^ The success of assisted reproductive techniques (ART) and outcomes of infertility treatment massively depends on the quality of sperm used. Many conventional techniques such as swim up, density gradient centrifugation, and magnetic activated cell sorting are being used to harvest high‐quality sperm fraction. However, such methods typically involve many steps, manual screening processes, and migration mechanism through complex environments, hence prone to human error and may also cause damage to sperm DNA.^2^, ^3^ Although, a new nano‐selection method (using magnetic nanoparticles to segregate apoptotic and acrosome reacted sperm fractions) proves to be a better alternative to conventional methods. But, a more recent promising and less invasive addition to sperm sorting techniques proves to be microfluidics. Microfluidics is the science and technology of accurate manipulation of fluids at sub‐millimeter scale, which is typically done in microchannels with dimensions of a few hundred micrometers.^4^ This technology selects sperms in a way similar to in vivo sperm selection within the micro confined areas in cervix and oviducts of female reproductive tract, when provided optimal temperature and the chemical environment.^5^, ^6^, ^7^ Not involving washing and centrifugation step as in other conventional techniques, microfluidics is least damaging to sperm DNA and feature least production of reactive oxygen species.^7^

Many live births have been obtained after artificial insemination in bovines following selection of highly motile sperm subpopulations with the help of microfluidics.^8^ The fluid dynamics in microchannel geometry and design aids in the propulsion of spermatozoa, thus ensuring ultrahigh‐throughput sorting. It uses microfabrication and sperm behavioral properties in vivo which aid in quality selection and better analysis.^7^ Moreover, microfluidic selection of functional sperm is characterized by sinuous trajectories, exhibiting many curves or turns during motion. Microfluidics target selection of undamaged motile spermatozoa which otherwise is a major limitation in conventional cell sorter sowing to vulnerability of physical damage.^9^ While selection of fertile sperm population for assisted reproduction in animals, advanced microfluidic chips have been tested to remove dead sperms and debris.^7^, ^10^ In bovines, more functional and progressively motile spermatozoa with high DNA integrity have been obtained,^9^ based on the ability of sperm to cross laminar flow streamlines. Highly motile spermatozoa with intact membrane integrity and mitochondrial function from frozen‐thawed bull semen have been successfully sorted using diffuser‐type microfluidic sperm sorter.^11^ DMSS is now considered by many as a high‐quality sperm selection method to achieve great potential of assisted reproduction in bovines. It is a well‐established fact that during in vivo conditions, there is facilitation of sperm migration with selection of small subpopulation of healthy and motile sperm which reach to the site of fertilization.^12^ It has been reported that mouse and human tract fluid allow rheotaxis‐based flow of sperm.^13^ Although many of these natural aspects are not achievable artificially but principles of novel microfluidic chips ensure laminar, unidirectional, or gradient flow in a 3D physical environment.^14^ These characteristics along with provision of altered chemical composition in the medium, make the procedure closer to natural processes and improve outcomes in in vitro fertilization.

While taking application part, microfluidics has become a tool to explore new possibilities within IVF technology.^15^, ^16^ Besides sperm selection, microfluidics aid in embryo culture, in vitro oocyte maturation, in vitro fertilization, and sperm processing, cumulus cell removal from zygotes, and in vitro microchannel insemination.^17^ This technology owing to lesser handling and manipulation of the gametes, poses lower risk of cellular damage resulting in greater success in IVF procedures. Intact sperm DNA is a key to successful fertilization, proper egg activation, and embryo development.^18^ Several studies indicate that the status of sperm chromatin at the time of fertilization can influence embryonic survival, thus, accounting for a significant proportion of male infertility. Besides other external sperm quality indicators, DNA integrity is now considered an important predictor of sperm fertility potential and IVF outcomes. Plethora of microfluidic based studies and reviews are of the opinion that microfluidic‐based systems facilitate noninvasive identification and quality selection of spermatozoa in terms of viability, functional capacity, morphology, and DNA integrity for in vitro fertilization. In this review, we focus more on the published literature in animals which supports microfluidics being the latest technology for quality sperm selection and analysis in humans as well as domestic and wild animals. According to reviewed research studies, the technique is so far considered novel, fast, reliable, more sensitive, and accurate even at the single sperm level. Our review also includes way forward and prospects of quality sperm selection and analysis for better outcomes in assisted reproduction. The advances in diagnostic and therapeutic evaluation of infertility based on selection of best quality sperm and sperm‐friendly semen analysis in domestic animals is also taken care.

## 2 HISTORICAL PERSPECTIVE IN THE DEVELOPMENT OF MICROFLUIDIC APPROACHES FOR ART

Roberts^19^ after studying motion of spermatozoa in fluid streams reported geotaxis behavior of sperm. The geotaxis was later reported to be rheotaxis behavior due to the response of spermatozoa to fluid shear and gravity.^20^ Discouraging production of harmful wastes in IVF media due to higher sperm oocyte ratio in human IVF (10000 sperms/oocyte) and considering the natural fluid flow that surrounds the gametes during fertilization, climbing‐over‐a‐wall (COW) method was first proposed.^21^ The method in porcine IVF allowed higher monospermic penetration rate after putting a wall between sperms and oocyte. Also, there was direct correlation of sperm concentration with oocyte penetration and an inverse correlation of sperm concentration with monospermic oocyte penetration. Later, straw method was devised to reduce the sperm concentration around oocytes and obtain sperm: oocyte ratios closer in vivo. Depositing semen at the one end and keeping oocytes at the other end of a 0.25 mL × 5 cm long semen straw, used as IVF channel, allowed lesser number of high‐quality sperms available for oocyte compared to standard drop protocol.^22^ Some researchers used polydimethylsiloxane (PDMS)/borosilicate microchannel for porcine in vitro fertilization and reported lower polyspermic and higher monospermic oocyte penetration using microfluidic device.^22^, ^23^, ^24^, ^25^ Some microfluidic device with dynamic media flow^26^ were used for embryo development with development of blastocyst stage in a duration similar to in vivo development. Later, spermatozoa were observed to control their direction by rheotaxis,^27^ chemotaxis, and boundary following behavior.^28^ Rheotactic behavior of sperm (upstream motion or movement against flow) was quantified recently using three‐inlet chip and collection chamber positioned between the spermatozoa and the flow inlet.^29^ The flow pattern was used in the chip to increase the total recovery rate of spermatozoa and progressively motile spermatozoa. Some successful designs of quality sperm selection microfluidic chips are those reported in porcine,^24^, ^25^ humans,^30^, ^31^ bovine,^8^ and mouse.^32^

## 3 WHY WE NEED MICROFLUIDIC BASED APPROACH?

### 3.1 Sperm heterogeneity

Semen samples contain a mosaic of sperm subpopulations possessing different patterns of motility and varied morphology. Some cells do have greater motility, some are more viable, some have better morphology, and others have more intact chromatin. We desire a population of sperms having all the attributes for greater outcomes in ARTs. Such populations possess qualities of highest fertilization capability and the best features for supporting embryo development. However, effective methods are lacking that lead to separation of specific high‐quality sperm subpopulation corresponding to that selected naturally in oviduct.^33^ Human spermatozoa in a sample has been shown to possess different characteristics in terms of motility, morphology, hyperactive motility, and protein tyrosine phosphorylation in response to incubation under capacitating conditions in different fractions of Percoll gradient.^34^ Jenkins et al. reported existence of differentially methylated regions in the DNA between high‐quality and low‐quality sperm fractions.^35^ While other study by these authors^33^ reported 15% of the ejaculate containing quality subpopulation migrating toward the higher temperature within a gradient had rhodopsin at a specific cellular location. Rhodopsins are G protein‐coupled receptor proteins in mammalian sperm cell which act as thermo‐sensors and trigger signaling pathways for thermotaxis.^36^, ^37^ A study on bull ejaculate by D'Amours et al.^38^ reported that sperm within two different sperm populations separated by density gradient centrifugation (DGC) showed that in high motile population, 80 proteins were in abundance compared to low motile spermatozoa where only 31 proteins showed abundance. Comet assay involving single‐cell analysis of DNA fragmentation revealed appreciable intra‐sample heterogeneity in human spermatozoa.^39^ DNA fragmentation ranged from 0 to 70% in normozoospermic men and from 0% to 65% in epididymal mouse spermatozoa.^37^ Differences in telomere lengths have also been reported by Antunes et al.^40^ in different sperm subpopulations. High‐ and low‐sperm chromatin compaction in different sperm subpopulations have also been observed.^41^ More comprehensive studies of semen ejaculate heterogeneity involving genome, epigenome, transcriptome, proteome, and metabolome studies may be the way forward to the selection of high‐quality sperms for assisted reproduction to improve outcomes and preserve quality genomes.

### 3.2 Polyspermic versus monospermic fertilization

Generally, many spermatozoa are required to be exposed to ovum in vitro to achieve fertilization out of which only a fraction of population is capable of fertilizing an oocyte. Several randomized controlled trials for the treatment of male subfertility show large variations in sperm oocyte ratios for achieving fertilization and the ratios mostly fall in the range of 50,000–10 million sperm/oocyte for IVF.^42^ However, latest studies show even lower limits of this ratio by selecting quality spermatozoa. A recent study in mouse achieved 60% fertilization rate using 5 motile sperm/oocyte by enhancing sperm capacitation via creatine supplementation in the human oviductal fluid (HTF) medium. Creatine in the medium increased sperm ATP production and motility.^43^ The concept of monospermic fertilization used in ICSI, dates back to COW method described in the historical perspective of this review. Natural oviduct secretions in mammals have been reported to contribute to increase monospermic fertilization through zona pellucida modulation and enhancing sperm–oocyte interaction and more power of penetration.^44^ Conventional methods of sperm selection hardly meet successful selection of a single sperm for use in assisted reproduction. However, more monospermic oocyte penetration was achieved in porcine oocytes using microfluidic device.^24^, ^25^ But comprehensive studies in this regard are lacking in literature.

## 4 CONVENTIONAL METHODS OF SPERM SELECTION

Conventional methods of sperm sorting include density gradient centrifugation (DGC), sperm washing, swim‐up (SU) and magnetic activated cell sorting (MACS), and MACS in conjunction with DGC.^39^, ^45^, ^46^ All these methods use repeated centrifugation and sperm flows in sheath fluid. Other important methods of sperm selection are hyaluronic acid‐coated slide sperm‐binding assay and electrophoretic approach. Several studies in humans and bovine reveal use of Percoll density gradient centrifugation, swim‐up, washing by centrifugation, glass wool filtration^47^, ^48^, ^49^, ^50^, ^51^, ^52^, ^53^, ^54^ for removing seminal plasma, dead cells, abnormal sperm, cryoprotective agents, and other factors for preparation of quality sperms.

Centrifugation technique at 200–1800 g with colloidal silica particles, results in appearance of distinct sperm bands separating motile sperms from immotile sperm and debris. However, the technique is associated with sperm mechanical damage, production of reactive oxygen species, and loss of DNA integrity which ultimately leads to lower fertilization rates, impaired embryo progression, and decreased pregnancy rates.^55^ More recent studies in human patients with mild or idiopathic male factor by Oguz et al.^56^ and animal sperm selection^57^ reported SU selected sperm population having less DNA fragmentation compared to its counterpart DGC. Also, the direct micro‐SU variant shows comparable fertilization percentages by ICSI to those of DGC. SU has been also associated with higher blastocyst development in vitro more pregnancy rates (42% vs. 26% in DGC) and low abortion rate (13% vs. 29% in DGC).^58^ MACS or annexin V‐MACS helps in separation of apoptotic and non‐apoptotic spermatozoa.^59^ It involves coating of magnetic nanoparticles with annexin V molecule^60^ and externalization of phosphatidylserine in apoptotic sperm in semen sample which isolates non‐apoptotic sperms with high DNA integrity.^61^, ^62^, ^63^ Varied results of comparison between MACS, SU, and DGC have been reported in various studies but a recent study in human sperm by Ziarati et al.^64^ reports more DNA integrity and embryo survival following MACS technique. MACS studies have not been carried out yet in livestock species. Rabbit sperms selected using AV‐MACS for AI reported no enrichment in non‐apoptotic spermatozoa and no influence on reproductive outcome.^65^ Hyaluronic acid (HA), a main extracellular matrix molecule surrounding the cumulus–oocyte complex (COC)^66^ bears binding sites for spermatozoa during spermatogenesis and maturation.^67^, ^68^ Comparison of HA‐binding assay with SU and DGC have contradictory findings but two separate studies by Parmegiani et al.^69^ and Huang et al.^70^ reported lower and similar level of DNA fragmentation in spermatozoa selected in HA solution compared to SU, and by HA‐coated dishes compared to DGC, respectively.

Electrophoretic approach is a more rapid isolation of motile, viable, and morphologically normal spermatozoa with good DNA integrity^49^ as revealed by high‐power microscopy.^71^ In fact, a negatively charged plasma membrane of spermatozoa forms basis for the developed methods of separation using electric field. It ensures selection of higher quality sperms characterized by high DNA integrity.^72^, ^73^, ^74^, ^75^ The charge‐based separation, also known as Zeta method, has allowed the selection of spermatozoa with lower DNA fragmentation compared to the HA‐coated dish selection.^73^ Although results by Zeta method being promising, only a single human study has been published using spermatozoa selected for ICSI^76^ and no study is available so far in animals. Other available conventional powerful cell sorters using flow cytometry are not suitable for fragile spermatozoa. Such sorters use florescence proteins, antibodies, and other compounds which are costly and the sorting process leads to greater mechanical damages and a recent study by Nakao et al.^32^ shows that in mouse, after flow cytometric sorting a smaller number of selected sperms obtained had tails moving up and down compared to sperm subpopulation obtained in microfluidic chip cell sorter in the same species. As reviewed in literature we found no established study regarding selection of quality sperm using flow cytometry. Methods, based on morphometric evaluation of spermatozoa^77^ and direct capture‐based spermatozoa selection with a low number of vacuoles and normal nuclear morphology under a microscope equipped with a micromanipulation system and a 6300X magnification^78^ have been used. But the techniques provided sperms which neither possessed high DNA integrity nor improved IVF outcomes and live births.^79^

Thus, none of the discussed techniques can isolate highly motile, morphologically normal sperm with high DNA integrity from an unprocessed semen sample. Also, variable results have been published in their outcomes after use in assisted reproduction. Moreover, many such techniques are costly, time consuming, and require more skill. All the methods bypass natural barriers that sperm would experience in vivo^80^ and damage sperm DNA in centrifugation steps^81^, ^82^ which has already been reported to have long‐term effects on viability of embryos.^83^ Besides, all these methods focus only on sperm motility as sperm quality metric. Sperm morphology and membrane properties are required to be explored for quality sperm selection and investigations regarding morphology as a sperm quality metric are not documented.

## 5 MICROFLUIDICS AND QUALITY SPERM SORTING

### 5.1 Physical aspects in microfluidics

Microfluidics refers to science and technology to manipulate small amounts of fluids accurately using microchannels of a few hundred micrometers dimensions.^84^ It helps in exploring different geometrical, sperm–fluid interaction, hydrodynamics of sperm, and other biochemical mechanisms in which sperm has to interact in order to attain motility to reach the site of fertilization. It is typically done in microchannels with dimensions of a few hundred micrometers. The technique uses microfabrication and sperm behavioral properties in vivo which aid in quality sperm selection. Microchannels so far successfully used include Y‐, H‐ and Radial Microarray‐shaped. These all are PDMS fabricated and coated with poly(ethylene glycol) methyl ether methacrylate (PEGMA). PEGMA is a nonlinear analog of polyethylene glycol (PEG). Moreover, each microscale microchannel is constituted by two inlet channels (for sperm and buffer medium), two outlet channels (selected sperm collection and waste collection), and a main channel.

The devices produce new insights of sperm behavioral changes enabling propulsive or progressive motility of quality sperms to be available for oocyte during in vitro production. The propulsion of sperm is attained in microscale environments through periodic but time irreversible changes in the shape of sperm's body (scallop theorem) and locomotory drag forces when viscous forces dominate inertial forces. The drag force is better explained by low Reynold's number (*R*_e_ = 4.9 × 10^−3^) of fluid dynamics.^85^ Low Reynold's number inertial forces are negligible while viscous forces dominate which causes a drag for locomotion. Drag force allows optimal laminar flow of semen and medium.

**FIGURE 1:**
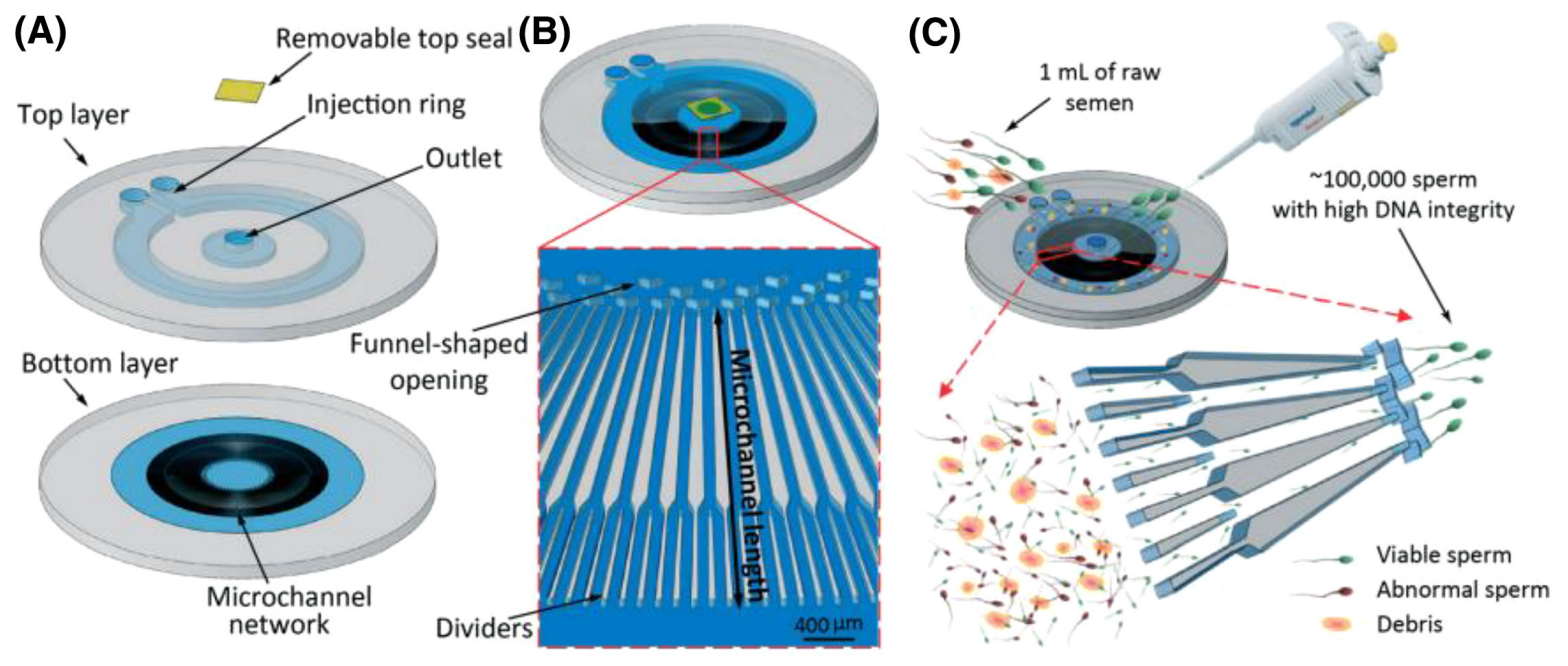
Microfluidic sperm selection device (capacity for one milliliter of raw semen). (A) Schematic view of the device: Bottom layer has a network of 500 radial microchannels with a 100 × 75 μm cross‐section, top layer (1.45 mm depth at the injection ring and 0.8 mm depth at the outlet chamber and transparent tape as the removable top seal). (B) Schematic view of the assembled device and close‐up view of the microchannel vertical walls. Each pair of consecutive dividers coalesces 5 mm before the outlet. Funnel‐shaped openings prevent sperm from re‐entering the microchannels after reaching the outlet. (C) One milliliter of raw semen is injected around the ring, live and motile sperm navigate through high viscosity fluid toward the central outlet, resulting in collection of ~100,000 sperm for ART. (Reproduced from Nosrati et al.^30^ with permission.)

A microfluidic chip with radial microarray design of microchannels was successfully used by Nosrati et al.^30^ for quality sperm selection in humans and bull (Figure 1). In this breakthrough design, the chip consisted of top layer having two inlets and an outlet with removable top seal. Buffer and semen samples injected enable streamlined flow of sperm through radial microchannel network (500 microchannels) on bottom layer. Each microchannel (400 μm long) has a funnel‐shaped opening and dividers. Out of a 1‐mL semen sample, 1,00,000 sperms with high DNA integrity were obtained in the study. Sperms were evaluated using florescent dyes.

## 6 INNATE PROPERTIES OF SPERM INSPIRING NEW METHODS OF ITS SELECTION AND ANALYSIS

### 6.1 Regulation of sperm motion

Locomotion of mammalian spermatozoa is driven and influenced by (1) mitochondria present in midpiece of tail or flagellum, generating force for beating of principle piece,^86^ (2) axoneme (9 + 2 + 2 pattern) at the central core of the flagellum, causing a wave‐like pattern through sequential sliding of the nine outer microtubule doublets via ATPactivated dynein arms over the neighboring doublet,^87^, ^88^ unidirectional flagellar wave owing to balance between active force of the dynein arms, passive force generated by flagellar and hydrodynamic drag force,^53^ and flagellar wave interaction with surrounding medium leading to drag force acting along flagellum which in turn results in drag anisotropy. The higher ratio of normal to tangent drag compared to normal to tangent velocity forms a propulsive force in the direction normal to the motion of the filament^89^ spermatozoa's helical or planar wave configurations^90^, ^91^ as a result of dynein motor regulatory mechanisms owing to changes in intra and extracellular conditions, ion flux changes, and share rate regulation.^92^, ^93^ The findings of the digital imaging studies revealed sperm's exhibiting wide range of swimming patterns starting from typical, helical, hyperactivated, hyper‐helical, and chiral ribbons. The sperm's typical swimming pattern characterized by forward progression with lateral displacement is observed in 90% population while ~5% of sperms swim along helical trajectories characterized by forward progress with a stable revolution. Swimming patterns are changed about every 10 s and ~2% of sperm exhibit a chiral ribbon swimming pattern characterized by forward progress with planar oscillation of the sperm head. Three‐dimensional shape and volume of a bovine sperm head was measured by Merola et al.,^94^ while using onchip holographic microscopy to quantify the volume of sperm cells. Using optical tweezers and a focused laser beam to generate an attractive force to trap and rotate the asymmetric sperm cell by regulating the laser power to record its holograms.^95^ Same author in 2010^96^ had reported 3D images of bovine sperm with detailed topographical information demonstrating a protuberance on the post‐acrosomal region of the sperm head.

### 6.2 Rheotaxis behavior of sperm and viscosity of medium

Natural in vivo mechanisms such as ciliary beating and surface secretion form viscosity gradients and flow which in turn influence sperm migration to the site of fertilization and the phenomenon is called rheotaxis.^13^, ^97^ Rheotaxis is an established property of sperm to swim against the flow.^98^ Two important rheological variables namely, viscosity gradient and flow rate are targeted to be achieved in microfluidics for quality selection. Viscosity determines the swimming trajectory of sperm as the increase in viscosity at a fixed energy production decreases flagellar wave frequency as well as wavelength hampering progressive motility. Further a combined viscous and elastic nature of mucus potentiates swimming velocity owing to formation of highly strained fluid regions behind the tail, responsible for inhibition of backward movements of sperm and enabling forward flow. Rheotaxis is exhibited in nonuniform flow field caused by a boundary^99^ and is also responsible for surface accumulation and sperm's boundary following behavior. Sperm has the capability to show rheotactic behavior in bulk fluid also (away from the surface), where weak shear flow is expected.^100^ Rheotaxis, a passive process, is a result of hydrodynamics^101^ and influenced by the front back asymmetry of the sperm.^99^ In the presence of a shear flow, both the higher resistive force experienced by the sperm head than the tail and drag forces from the flow over the conical envelope of the flagellar wave reorient the sperm against the flow, directing movement upstream. Besides vast human sperm studies, Kantsler et al.^98^ studied rheotaxis within bull sperm samples in circular crosssection microchannels. A combination of increased shear rates near a no slip boundary, conical shape of the flagellar wave envelope, and chiral flagellar beat pattern were observed to contribute to the upstream navigation of sperm with a spiral trajectory component around the circular channel circumference. Rheotaxis, in combination with boundary following behavior, has been shown to act as a long‐range guidance mechanism for sperm. Rheotaxis in bull semen has established studies in literature. A recent study by El‐sherry et al.^102^ reported maximum rheotaxis behavior of bull at pH 6.4–6.9.

### 6.3 Chemotaxis behavior of sperm

In vivo environment of female reproductive tract provides a characteristic chemotaxis attracting sperm toward egg in response to intercellular mechanisms generating a chemical signal.^103^, ^104^, ^105^ Such studies are available in more detail in case of sea urchin sperm.^106^, ^107^ The studies explain react gradient (continuous release of chemoattractant peptide by the egg thereby stimulating sperm motility, inducing intercellular alkalization and polarization), activating the sperm‐specific Ca^2+^ channels (CatSper), and ultimately raising intracellular Ca^2+^ concentration inside the cell.^108^, ^109^ More Ca^2+^ leads to increase in flagellar beating, aligning the sperm swimming trajectory and guiding sperm toward the egg. The sperm chemotactic mechanism in sea urchin predicts successful fertilization in vivo.^110^ However, in mammals the chemotactic response of spermatozoa has been challenging due to unresolved chemotactic role and chemotactic intercellular mechanisms.

More advanced rapid microfluidic technologies capable of single cell analysis such as droplet based microfluidic technologies show a high‐throughput response of individual sperm to a chemical cue and promise better study of chemotaxis in a precisely controlled and isolated environment. Koyama et al. ^111^ and Xie et al.^15^ studied the chemotactic response of mouse sperm in presence of ovarian extracts and cumulus cell masses near diffusion chamber of the microfluidic devices. The studies indicated progesterone as chemoattractant for meager sperm population (7% and 10% of sperms, respectively). However chemotactic role of progesterone was not observed in later studies of mouse like hydrogel based microfluidic chemotaxis plate form used by Chang et al.^112^ did not show any chemotactic response of mouse sperms in presence of progesterone. A contradictory response in more recent microfluidic study of Zhang et al.^113^ indicated progesterone acting as chemoattractant at 1 mM concentration gradient but not at 100pM gradient for human capacitated sperms. Thus, the chemotactic role of progesterone and chemotaxis behavior of sperm needs more controlled studies which can better ensure using controlled microenvironment in microfluidics.

## 7 MICROFLUIDIC DESIGNS

### 7.1 Basic microfluidic system

A basic microfluidic system consists of the main microchannel with two inlets and two outlets. Poly dimethyl silicone (PDMS) has been used as a fabricating material for construction of these devices due to it being non deleterious to sperm survival, fertilization, and early development of the embryo.^114^, ^115^, ^116^ The device dimensions (length, width, and height) are specific to the size of sperm and species. In general, for human spermatozoa, they are 5 mm, 400 μm, and 50 μm, respectively in a K‐shaped device. The diameters of the inlet‐up are smaller (100 μm) than inlet‐down (300 μm), and outlet up and outlet down are same in size as that of inlets. Semen is injected into microchannel from the inlet up, motile sperms varying with laminar streams get pre collected inlet‐down due to its larger width than that of the inlet‐up. The height of the microchannel needs to be lesser than length of spermatozoa in order to allow its unidirectional flow, preventing z directional movements to improve sorting efficiency. Maintaining optimum viscosities of semen and media and the flow velocities of the two inlets (300 μm/s) helps in uniform distribution of concentration by simulation. Fluorescent staining as used by Nakao et al.^32^ in mouse sperm and a statistical method based on flow cytometric analysis^117^ are employed to characterize both live and dead sperms and to analyze the sorting efficiency of motile sperm.

### 7.2 Microfluidic chips

Microfluidic systems are also named as Micro‐Total Analysis Systems (μTAS) or lab‐on‐a‐chip (LOC) devices. Apparent random motion of sperm in a quiescent medium, leading to diffusion is used within space constrained microfluidic channels to separate racing sperms. Persistence length of collected sperm was increased in a similar design by addition of pillar arrays.^118^ Microfluidic devices, typically fabricated by microfabrication techniques, enabling functional microscale parts for delivery and mixing of fluids^119^ along with separation of fluid particles.^120^ Nagata et al.^8^ used a bovine microchip to select microfluidic sorted sperm for live births in AI (diffuser‐type microfluidic sperm sorter). The chip was fabricated by micromachining of the poly methyl methacrylate (PMMA) master and replica molding using poly dimethyl siloxane.^121^

Some other important microfluidic chips used so far in sperm selection documented in literature include Sperm check, FertilMARQ Fertile Plus and Fertell in humans, on Chip Sort, on Chip Biotechnologies, Japan in mouse, and Fertile Bovine and Fertile Plus®, KOEK EU GmbH, Hannover, Germany for bull semen. SpermCheck,^122^ a paper‐based microfluidic device, can evaluate lowest sperm count even below the WHO reference value (<20 × 106 mL^−1^) with 100% concurrence for diagnosis of low‐quality sperm. The commercially available test Fertell can detect semen samples with low motility.^123^ FERTILE® chips are being used in IVF, IUI (intra uterine insemination), and ICSI, providing selected sperm with best DNA and physiological quality in less time. FERTILE Bovine® product mimics more to female reproductive tract system and this technology selects sperm based on natural sperm movement.

**FIGURE 2:**
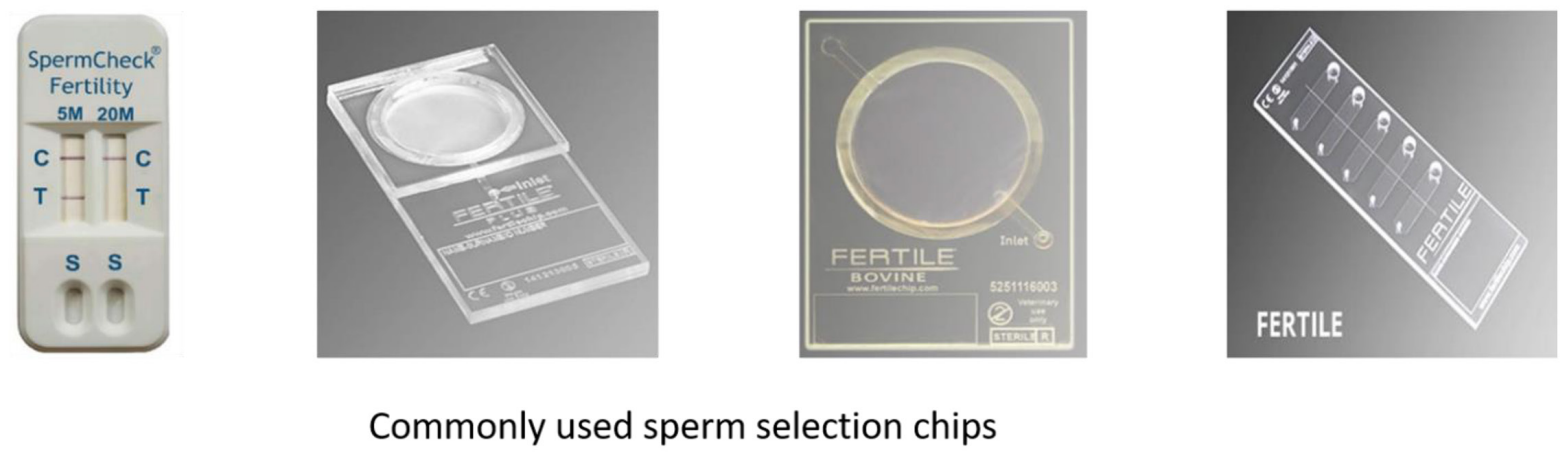
Common microfluidic chips used in semen laboratories for sperm selection.

Common microfluidic chips used for sperm selection is shown in Figure 2.

### 7.3 Main functions of microfluidic devices

The devices have ability to sort sperm cells in a faster and easier way, closely mimicking the natural selection processes. Besides their role in analytical chemistry, molecular and cellular biology, microbiology, and pharmaceutical drug screening, these devices ensure more sample manipulation processes associated with cell culturing, cell separation, and DNA analysis. Moreover, the unique microscale properties of microfluidic devices have been successfully employed in assisted reproductive technologies (ART) for sperm sorting,^124^ oocyte manipulation,^125^ insemination,^126^ embryo culturing,^127^ and for assessing sperm and embryo quality.^128^ The devices can select spermatozoa having greater motility, viability, morphology, and DNA integrity.^5^, ^30^, ^126^, ^129^ Besides the technology in animals reduces the content of reactive oxygen species (ROS) of sperm and the potential for DNA damage,^130^ compared to conventional methods. Microfluidic sperm sorting chips show greater efficiency for bull sperm; however, investigation is still required for its ability to increase embryonic development rates and IVF outcomes in bovine. A recent study by Gonzalez‐Castro et al.^131^ to select sperms on the basis of sperm motility and morphology using microfluidic chip in horses demonstrated the technology as the only sorting method to get subpopulation of sperm with greater DNA integrity. In this study, equine frozen‐thawed semen was subjected to sorting, using human microfluidic chip (FERTILE PLUS™ Sperm Sorting Chip, DxNow Inc.) with modifications in manufactures guidelines. Although microfluidic chip did not improve percentage of motile, live, morphologically normal, and swollen spermatozoa as compared to single‐layer colloidal centrifugation (SLC) and swim up (SU) sorting methods but the percentage of DNA‐fragmented sperms was significantly reduced (6% in microfluidic sorting vs. 12% in SLC and 11% SU). However, advantage of using microfluidic sorting in horses for clinical ICSI is still to be determined. Microfluidic systems help in selection of qualitatively and quantitatively sufficient sperm and reduce requirements of clinician's skill for sperm purification process. Use of mechanical conditions in micro fluidics,^127^ tiny culture drops and micro well approach^132^ lower possibility of dispersion of autocrine factors, which remains unidentified yet. Due to its resemblance to fallopian tube environment and providing natural boundary following navigation, rheotaxis and random motion in microchannels, it remains an advanced method of choice for sperm selection.^133^

Healthy mouse off springs following IVF using high‐quality sorted sperm through microfluidic chip was reported by Nakao et al.,^32^ which indicates a mile stone in the microfluidic sperm sorting. The authors reported that fluoroscein‐5‐isothiocyanate (FITC)‐labeled peanut agglutinin (PNA) sperm sorted in the chip had good motility (> 50 μm/s progressive motility), fertilization, and developmental abilities (nearly 65% in highly acrosome reacted sperm and 100% for two cell‐stage embryos). Moreover, 30% live pups were obtained out of transferred embryos after IVF. Also, same study reports selection of acrosome reacted sperm having high fertilization rate following capacitation like changes in microfluidics. Such type of successes are yet awaited in other species following advanced microfluidic sperm selection procedure. Further microfluidics can be an innovative approach of sperm selection to improve IVF outcomes in mammals.

## 8 SPERM SELECTION BY MICROFLUIDICS

Sperm sorting by microfluidic uses microfluidic devices that isolate only motile sperm, referred as Type 1 microfluidic sperm sorting. Microfluidic devices isolating sperm cells without relying on sperm motility form Type 2 sorting, and the devices for observation and selection of individual sperm constitute Type 3 microfluidic sperm sorting. All three types have their characteristic features and separate clinical applications.

Type 1 sperm sorting devices constitute a largest group including technologies translating the process of motility screening to a microfluidic system, thus improving the swim up method. Type 1 microfluidics have ability to select enough motile sperm cells (>10 million cells) for intrauterine insemination (IUI) and pure and lesser in number (~100,000 cells) for in vitro fertilization (IVF) procedures. The sperms sorted with these systems are almost 100% motile and with acceptable morphology and DNA integrity. Type 1 sorting is a natural way to separate sperms from somatic cells and debris in semen. Moreover, dead and damaged sperm cells are also segregated. In type 1 system, motile sperm subpopulations are selected after exposure of semen sample to laminar stream‐based, surface‐modified microchannel microfluidic chips.

Type 2 microfluidics instead of using sperm motility; utilize shape, size, or other physical biomarkers of sperm as a selection criteria. Trapping of sperm in these systems are focused to retain the full fertilization capability of a subfertile semen sample by indiscriminately capturing sperm cells. Hence, the selection mechanism is not explored to sort an improved sperm subpopulation.

Type 3 microfluidic devices are employed to capture and non‐invasively investigate the characteristics of a single sperm cell without affecting its viability. Such devices use Raman spectroscopy in combination with microfluidic sperm sorting systems. Raman spectroscopy is a vibrational spectroscopy involving inelastic scattering of monochromatic light by the molecular structure of a system to determine its constituents. Individual viable sperm cells have Raman spectras with common traits, leading to identification of sperm biomarkers and measurements of sperm DNA and organelle damage.^134^

Some of the technologies and their applications in semen selection are briefly summarized in Table 1.

**TABLE 1:** Some remarkable microfluidic‐based technologies and their applications in sperm selection for assisted reproduction.

Microfluidic device	Design/principle	Species	Outcome	Reference
H‐Filter microfluidic device	H design filter with three inlets and three outlets, only motile spermatozoa to cross the streamlines in microfluidic channel. Continuous stream instead of batchwise	Human	Retrieved motility 40% Velocity of the liquid is the highest in the center of a channel	[114]
Rheotaxis‐based microfluidic device	Used the tendency of spermatozoa to swim against the flow and made use of this in their device to isolate the motile ones. Only two inlets	Human	Sorting rate of approximately 11 units per minute Easy handling of the chip	[27]
Chemotaxis‐based device	7 mm long Y‐channel that connected three wells. Cumulus cells placed in one well to act as chemoattractant	Humans	10% sperms show chemotactic behavior Chemotactic index of 1.25 which corresponds to ~55% cells displacing toward and 45% cells away from chemotactic signal	[15]
Boyden chamber type Thermotaxic device	A shallow temperature gradient (0.014°C/mm) across a tube, and a stainless steel porous membrane separating two compartments. spermatozoa placed in one chamber accumulate across the membrane in the other chamber	Humans	With increasing gradient larger accumulation of sperms helped in sorting	[164]
Themotaxic microfluidic chip	Consisted of two collection chambers, an interfacial valve to close off, trapping different spermatozoa in their respective chambers	Humans	Thermotactic reaction was observed in 10% of the sperm populations	[165]
Microfluidic chip	500 microchannels in parallel, which are connected to an inlet ring and an outlet in the center of the chip	Human	DNA fragmentation index could be lowered by a factor of 5–10 times	[30]
Microfluidic channel with different swimming medium	Device used controlled environment hyaluronic acid and methylcellulose as viscosity medium	Human	Hyaluronic acid was detrimental to the viability and motility of the spermatozoa when compared to methyl cellulose	[69]
Microfluidic chip	In‐ and outlet with a straight channel in between Hexagonal pool of 4 mm diameter with six adjacent channels with a width and height of 700 μm by 50 μm, connected by microchannels of 5 μm by 2 μm to the main pool Chemoattractant progesterone at 100 pM and 1 mM allowed to diffuse in microchannels	Human	Normal spermatozoa count 27.1% spermatozoa that swam toward the gradient was ~16% larger	[113]
Swim‐up method based transwell system	Device connected to porous membrane, allowing swim up	Human	Selected sperm morphology >60% instead of 20% in swim up Motility 90% instead of 40%	[118]
Thermotaxis based microfluidics	A drop of a medium containing spermatozoa connected to second drop with no cells. Under a temperature gradient. Spermatozoa respond by thermotaxis shifting toward higher and accumulate in second drop	Bull	Bull spermatozoa show a greater response to thermotaxis and achieve acceptable pregnancy following artificial insemination	[166]
Rheotaxis base chip	Designed with Serial of microchannels directed toward a well In response to the flow the spermatozoa swim toward it passing through the microchannels and accumulating in a receptive well where they can be collected for downstream applications	Bull	Level of DNA fragmentation only 0.37% compared to unselected semen (7%) 20 times lower dose of frozen bull sperm achieved acceptable pregnancy following artificial insemination	[8]
Chemotaxis based Sperm Selection Assay (SSA) device	Progesterone gradient was used for separation	Bull	Quality bull spermatozoa separated. Improving cleavage rates using sexed and unsexed semen for IVF	[167]
Thermotaxis based microfluidics	A drop of a medium containing spermatozoa connected to second drop with no cells, under a temperature gradient	Mice, humans	Spermatozoa respond by thermotaxis shifting toward higher and accumulate in second drop High DNA integrity Higher cleavage implantation and live birth rates	[33]
Chip using combined thermotaxis and chemotaxis	Combined chemotaxis chip with an on‐chip heater	Humans	Almost same number of spermatozoa moved toward upstream gradients in both mechanisms	[168]
Microfluidic chip	PDMS chip, 50 μm high, three 100 μm inlet channels merging into a 300 μm wide main channel and split into three outlets Sandwich structure, inner channel used to introduce the spermatozoa and the two outer channels having equal All immotile spermatozoa flow into the waste channel	Boar, Bull	Sperm viability decreased only by 6% and in case of bull viability remained almost unaffected compare to flow cytometric analysis	[169]
Microfluidic chip cell sorter On‐Chip Sort, On‐chip Biotechnologies, Japan	Pulsed air pressure, which results in minimal damages to cells Epididymal sperm sample dissolved in calcium‐enhanced human tubal fluid (mHTF) were sorted using a signal distribution of forward scattered light (FSC) and side scattered light (SSC) Sperms gated into scatter, labeled with fluoroscein‐5‐isothiocyanate (FITC) and collected in the collection reservoir	Mice	Selected acrosome reacted spermatozoa showed in vitro fertility and full developmental ability	[32]
Micro‐chamber‐based microfluidic platform	Traps nonprogressive sperm in microchambers separate, separating progressively motile sperm from	Bull	a DNA integrity of selected sperm improved by 20% b Average‐path velocity of the motile sperm increased from 57 ± 10 μm/s to 81 ± 13 μm/s a A high‐throughput, robust, fast (<10 min) and efficient, collection of progressive sperm	[170]

## 9 MICROFLUIDIC BASED SPERM ANALYSIS

Microfluidic sorted sperms are subsequently assessed for kinematic parameters, acrosome reaction, mitochondrial membrane potential, and DNA integrity. Notable parameters evaluated before use being sperm viability, motility, and morphology. But DNA integrity is now considered in latest technologies to improve in vitro production results. Semen analysis helps in quantifying sperm deficiencies like count, vitality, motility, morphology, and DNA integrity,^135^ thereby aids in diagnosis and treatment of male‐factor infertility^136^, ^137^, ^138^, ^139^, ^140^, ^141^ and efficient sperm selection. Available clinical methods^140^, ^142^ in humans utilize WHO reference values for semen characteristics.^137^ Conventional clinical methods include counting chambers, viability assays, Computer‐Assisted Sperm Analysis,^140^, ^143^ and DNA integrity assays (such as the comet assay and sperm chromatin structure assay,^144^, ^145^). However, being time consuming (counting chambers, viability assays), costly (CASA), more skill demanding, and low‐standard procedures (CASA, COMET and SCSA),^141^, ^146^ these methods are discouraging for improving efficiency in assisted reproduction techniques. Counting chambers method involves use of hemocytometer and Makler chambers for counting. Swelling of live sperm cells due to an influx of water to their cytoplasm from an induced osmotic pressure gradient of a hypotonic solution better distinguishes live sperm population. Although DNA integrity tests (SCSA and COMET) assist in furnishing detailed information about sperm chromatin characteristics, compaction and dis integrity, but wide applications of these techniques is also limited owing to less instrumental access, lack of standardized methods, and nonavailability of reference values for clinical evaluation.^141^This review therefore concludes that there is a gap to comprehensive regrading availability of most efficient semen analyzing techniques in order to prepare a best quality sperm to the oocyte in in vitro conditions. This way assisted reproductive outcomes might be closer to natural in vivo reproduction. Chen et al.,^147^ were able to calculate sperm concentration in the range of 0–252 × 10^6^ mL based on sperm's random swimming orientation principle in microfluidic chips. De Wagenaaret al.^148^ in a glass microchip with two sets of electrode gates and induced fluid flow based on electric impedance could estimate sperm abnormalities to the extent of 89%. A recent successful study by Nosrati et al.^31^ reported sperm concentration in the range of 8.56–381 × 10^6^/mL, motile sperm motility in the range of 3.73–315 × 10^6^/mL, and sperm motility ranging from (9 to 87%), using calorimetric signal principle in a fast run (10 min) paper‐based microchip.

Compared to conventional semen analysis, processing damages to human and animal spermatozoa in microfluidic designs are least observed in several reviewed research studies. The techniques are now regarded as accurate, fast, and easy to use but need comprehensive comparative evaluation.

## 10 MICROFLUIDIC‐BASED SPERM RECOVERY

Isolation of nonmotile sperm from background contaminated fraction in testicles has not been studied comprehensively. Magnum et al.^149^ have reviewd some studies of sperm testicular extraction but the methods prove less efficient. Microfluidics recovery of sperm from biopsy tissues, testicles, or epididymis proves faster and contamination‐free technique. This has been successfully demonstrated through the application of 3D printed microfluidic hard chips to recover sperm from mixed cell suspensions with a >96% recovery rate.^150^ This helps in sperm isolation from background cells and debris without affecting its vitality, motility, morphology, or DNA fragmentation to improve ART outcomes.

## 11 DNA FRAGMENTATION IN MICROFLUIDICS

Selecting quality sperm needs the ejaculates to be screened better for separation of non‐DNA‐fragmented or least DNA‐fragmented sperms available for oocytes in invitro studies. DNA fragmentation in microfluidic devices is lower thereby improves fertilization, and zygote and embryo developments. Shirota et al.^5^ while studying separation efficiency of a microfluidic sperm sorter reported very low DNA damage in selected sperm subpopulation. In fact, the selected spermatozoa exhibited 95% motility, and only 1% DNA fragmentation index. In a more recent study by Pérez‐Cerezales et al,^33^ authors reported a range of 0%–70% DNA fragmentation of individual spermatozoa in normozoospermic men and 0%–65% in epididymal mouse spermatozoa. Gai et al.,^151^ while using acoustic based continuous‐flow microfluidic method for bull sperm selection, found it capable of selecting high‐quality sperm with considerably improved motility and DNA integrity compared to the initial raw bull semen. The method was reported to select sperm having improved motility, progressive motility and DNA integrity up to 50%, 60%, and 38%, respectively. Using SCSA, TUNEL, and SCD, different studies have shown lower DNA disintegrity in microfluidic chips; some recent and important findings are presented in Table 2.

**TABLE 2:** DNA fragmentation in microfluidic chips versus conventional sperm sorting techniques.

S No.	Device	DNA fragmentation (%)	Reference
1.	Microfluidic radial 500 channels in parallel	2.4 vs. 10.9 (control)	[30]
2..	Sperm sorter quails	5.9 vs. 8.3 (SU) and 27 (control)	[152]
3.	Sperm sorter quails	0.8 vs. 10 (DGC‐SU)	[5]
4.	Microfluidic DMSS (Bovine)	0.37 vs. 7.08 (unsorted)	[8]
5.	Fertile (Zymote) device	0 vs. 15 (DGC‐SU)	[153]
6.	Simple Periodic Array for Trapping and Isolation (SPARTAN)	4–6 vs. 13 (SU)	[118]

## 12 ROLE OF MICROFLUIDICS IN IVF

In addition to role played by microfluidics in quality sperm selection and analysis, it helps in oocyte's maturation, enucleation, transport, and cryopreservation. It also helps in development and culture of in vitro produced embryos owing to improved cleavage rates (67% vs. 49% in standard procedures).^154^ Studies have indicated that oocytes maturated in microchannel had the same nuclear maturation of those maturated in standard drops. Microchannels used for maturation of individual oocytes enhance developmental potential and aids in transporting the oocytes in the microchannel up to a specific point (trap) where the fertilization is carried out.^155^. Zeringue et al.^156^ first used microfluidics to remove cumulus cells of bovine oocytes and found microfluidic procedure more efficient and less stressful compared to vortexing for cumulus removal. The study reports development to more advanced stages of 2 days embryo was higher in the microfluidic device than in the control treatment (35% vs. 20%) and obtained more blastocyst (57% vs. 30%).

Yuan et al.^155^ reported increased developmental potential of embryos using silicon microchannels probably due to oocyte movement with the help of pumping action of medium over the oocyte.^125^A recent study by Caliscici et al.^157^ used sperm cells selected after thawing at 30°C by standard density gradients (DG) protocol (SpermFilter®, GYNEMED GmbH & Co. KG.) and a microfluidic sperm sorting (MSS) chip technique (Fertile Plus®, KOEK EU GmbH). The authors observed more embryonic development rates and outcomes in routine IVF procedure using MSS chip‐based sperm (Day 8 blastocysts rates, 33.6% vs. 22.0%, using MSS chip sorted and DG sorting sperms, respectively). Also, Caliscici et al., 2019^157^ reported in IVF using microfluidic sorting chips (FERTILE PLUS), more blastocyst development rates (18.1 vs. 15.3% in MSS chip and DG, respectively) and cleavage rates (75% vs. 71.2% in MSS chip and DG, respectively). However, the extensive studies are lacking in this regard.

## 13 CONCLUSIONS AND FUTURE PERSPECTIVE

In spite of availability of various methods of sperm selection, conventional capillary zone electrophoresis selecting robust spermatozoa without damaging DNA^158^ and microfluidics are the best selection techniques. Microfluidics is a fast technique for selection of high‐quality spermatozoa but its full potential species wise has not been fully realized. Microfluidic selection improves percentage of normal morphology, viability, and DNA integrity in quality subpopulations spermatozoa to be used in assisted reproduction. The micro environment owing to small‐scale dimensions and controlled flow of the fluid in microfluidic chips aids in selection of sperms lower in number but higher in motility, DNA integrity, and fertilizing capacity, mimicking natural selection mechanisms. The devices also aid in studying spermatozoa's different behavior types in groups as well as investigating single sperm cell characteristics. Moreover, recent development in microfluidic technology designs led to encouraging outcomes in in vitro studies. Microfluidic sperm separation can be used to conserve wildlife germplasms and the methods devised for wild animal species can have large application in other domestic animals.

Commercialization problem in such devices limits applications in humans as well as animals. High cost of PDMS for fabrication of chips is another limitation for wide application in masses. Thermoplastics based microfluidic designs have been used with varying results in published literature. Similar to sperm robotics technique of sperm selection, reviewed in Bhat et al.,^159^ 3D printing facility in microfluidic designs,^160^ with the help of thermoplastics and other polymers for rapid prototyping enables faster commercialization and overcoming limitation for microscale.^161^, ^162^, ^163^ Majority of the studies found in literature are human side. Among animals only few bovine and porcine studies using microfluidic designs for sperm selection have been published. However, for better clinical adoption of microfluidic systems more user friendly, simple and robust designs need to be devised in humans and their modifications need to be investigated in order to apply these techniques in domestic and wild animal germplasm conservation. The sperm behavioral characteristics in vivo need to be further investigated and used as guiding physics for designing new and innovative microfluidic designs in all the species. The species‐specific microfluidic chips need to be designed for extraction of fertile spermatozoa with minimum DNA damage for use in assisted reproduction.

## AUTHOR CONTRIBUTIONS

The first and second authors conceived the ideas presented in the manuscript. The first author collected literature and wrote the manuscript. The second and third authors reviewed the entire manuscript.

## DISCLOSURES

The authors declare no conflict of interest.

## Data Availability

This is a review of literature. There are no new primary data presented in the manuscript, it is all cited and published in data repositories like CORD and DOI. Data sharing is not applicable.
